# Adaptive power-controllable orbital angular momentum (OAM) multicasting

**DOI:** 10.1038/srep09677

**Published:** 2015-05-19

**Authors:** Shuhui Li, Jian Wang

**Affiliations:** 1 Wuhan National Laboratory for Optoelectronics, School of Optical and Electronic Information, Huazhong University of Science and Technology, Wuhan 430074, Hubei, China

## Abstract

We report feedback-assisted adaptive multicasting from a single Gaussian mode to multiple orbital angular momentum (OAM) modes using a single phase-only spatial light modulator loaded with a complex phase pattern. By designing and optimizing the complex phase pattern through the adaptive correction of feedback coefficients, the power of each multicast OAM channel can be arbitrarily controlled. We experimentally demonstrate power-controllable multicasting from a single Gaussian mode to two and six OAM modes with different target power distributions. Equalized power multicasting, “up-down” power multicasting and “ladder” power multicasting are realized in the experiment. The difference between measured power distributions and target power distributions is assessed to be less than 1 dB. Moreover, we demonstrate data-carrying OAM multicasting by employing orthogonal frequency-division multiplexing 64-ary quadrature amplitude modulation (OFDM 64-QAM) signal. The measured bit-error rate curves and observed optical signal-to-noise ratio penalties show favorable operation performance of the proposed adaptive power-controllable OAM multicasting.

In general, there are two forms of angular momentum for a light beam: the first associated with polarization called spin angular momentum (SAM)^1^, and the second related to spatial phase distribution named orbital angular momentum (OAM)^2^. When a light beam is circularly polarized, each of its photons carries an SAM of 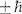, where 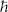 is the reduced Planck constant and the sign is positive for left-hand and negative for right-hand circular polarization. So for an SAM-carrying beam, there are only two states corresponding to left- and right-hand circular polarization. While for an OAM-carrying beam with a helical phase front, characterized by an 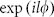 azimuthal phase dependence, the OAM has the discrete value of 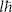 per photon where 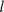 is called orbital angular quantum number or topological charge number. The topological charge 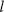 *,* in principle, can take arbitrary integer number ranged from 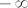 to 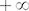, therefore, the state of OAM-carrying beams is infinite in theory. OAM-carrying beams, also called OAM beams, have been widely used in a variety of interesting applications, such as microscopy, optical manipulation, particle trapping and quantum information processing^3^,^4^,^5^,^6^,^7^,^8^.

Recently, OAM beams have gained much interest for increasing transmission capacity and spectral efficiency in communication systems^9^. Different OAM beams can be employed as information carriers for spatial mode-division multiplexing due to their intrinsic orthogonality. Communication with OAM (e.g. OAM multiplexing) has made huge progress in both free space and fiber transmission systems^9^,^10^,^11^,^12^,^13^,^14^,^15^,^16^, providing a potential alternative to alleviate the emerging capacity crunch.

In a multiplexing system, different channels usually carry different data information. However, there are many scenarios in today’s networks that require the multi-copy replication of a seed optical signal, also known as optical multicasting. Some of those scenarios include video distribution, teleconferencing, interactive distance learning, distributed games, live auctions, distributed computing and so on. Multicasting in the wavelength domain, i.e. delivery of an optical message to different wavelengths, has been performed in all-optical fiber-based systems and on-chip integrated devices^16^,^17^,^18^. All-optical wavelength multicasting supporting point-to-multipoint connections directly on the optical layer is of great importance for reconfigurable wavelength-division multiplexing (WDM) networks for alleviating the data blocking and improving the efficiency and performance. Similar to the widely used wavelength multicasting in WDM systems, all-optical mode multicasting may see potential use in mode-division multiplexing (MDM) networks. Very recently, 1-to-7 and 1-to-34 multicasting of OAM modes with phase-only spatial light modulators (SLMs) have been experimentally demonstrated^19^,^20^. The power of each multicast OAM channel is designed to be equal in these previous works. However, the actually measured back-conversion (from OAM mode to Gaussian-like mode) power distribution of the multicast OAM channels somehow deviates from the theoretical assumption. This is because different OAM modes feature different back-conversion spot sizes. Therefore, it is valuable to develop an improved scheme to correct the deviation between the experiment and theory. Moreover, one would also expect to flexibly control the power of each multicast channel considering the fact that different users (different channels) may require different power in practical applications. In this scenario, a laudable goal would be to develop a simple and scalable approach to adaptively and arbitrarily control the power of each multicast OAM channel, which has not yet been reported so far.

In this paper, we experimentally demonstrate an adaptive power-controllable OAM multicasting scheme by introducing a feedback process. The input data-carrying Gaussian mode is spatially modulated by a complex phase pattern loaded on an SLM to generate multiple data-carrying OAM modes for OAM multicasting. The power of each multicast OAM channel can be arbitrarily controlled by designing and optimizing the complex phase pattern through the adaptive correction of feedback coefficients. Moreover, power-controllable OAM multicasting carrying orthogonal frequency-division multiplexing 64-ary quadrature amplitude modulation (OFDM 64-QAM) data signal is demonstrated in the experiment showing favorable performance.

## Results

### Concept of adaptive power-controllable OAM multicasting

**Figure 1:**
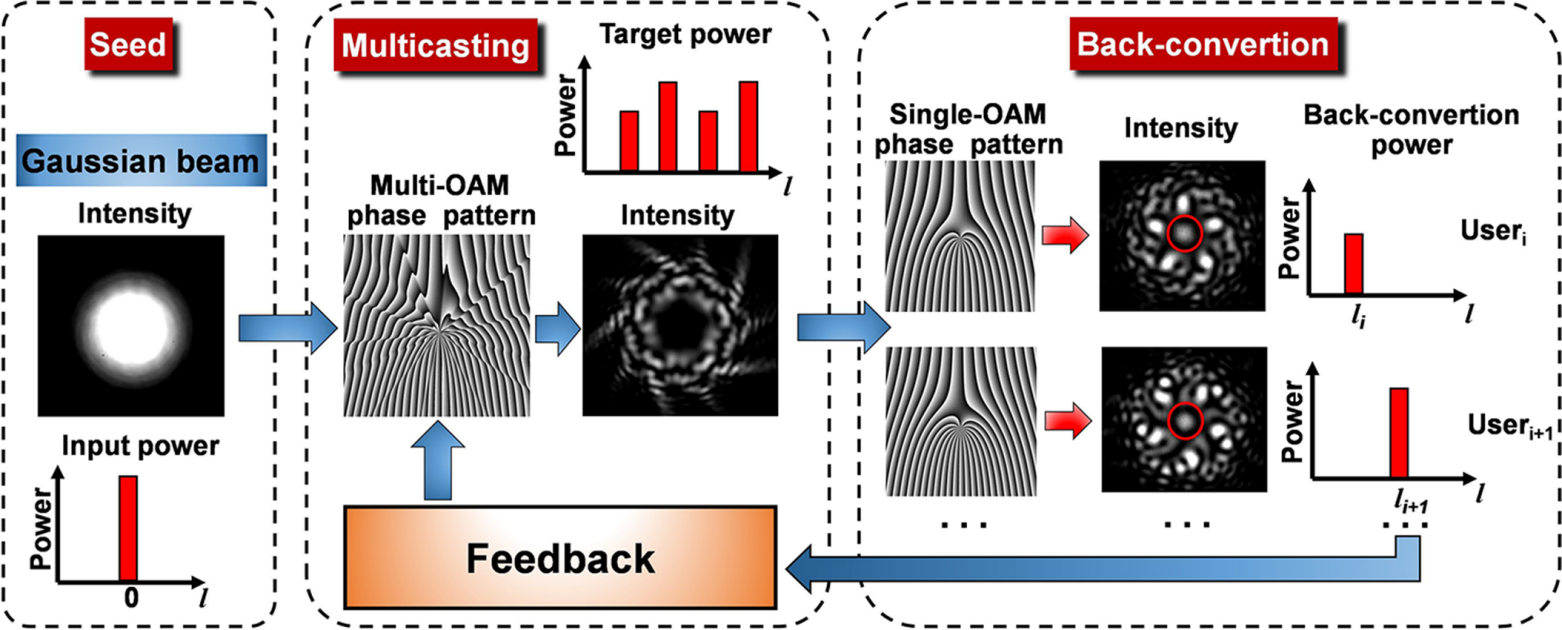
Concept of feedback-assisted adaptive power-controllable OAM multicasting.

The concept of adaptive power-controllable multicasting of OAM modes is shown in Fig. 1. An input Gaussian mode ( 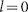) passes through a multi-OAM phase pattern generating collinearly superimposed multiple OAM modes with the power of each OAM mode determined by the phase pattern. In order to achieve target power distribution of multicast OAM channels, a specific complex phase pattern is usually prepared. However, the actually received back-conversion power of each multicast OAM channel could be offset from the designed target power distribution. This can be explained with the fact that a practical receiving system may have different receiving efficiency for different OAM modes having different back-conversion mode sizes. To make the received back-conversion power distribution more close to the target power distribution, a feedback is introduced to assist the generation of complex multi-OAM phase pattern with specific target power distribution. The feedback firstly gets the back-converted power of each OAM channel, and then computes the power difference between the measured power and target value. According to the calculated results, a new optimized complex multi-OAM phase pattern is created to correct the deviation between the experiment and theory. Repeat the similar feedback process until the measured power distribution is equal to the target one. Consequently, data information carried by the input Gaussian mode is copied and delivered to multiple distinguishable orthogonal OAM modes with expected target power distribution. Those multiple OAM modes can be distributed to multiple users. Due to the existence of a phase singularity of OAM modes, there is no intensity at the center of superimposed multiple OAM modes. For the back conversion from OAM mode to Gaussian-like mode, each user can use a different inverted single-OAM phase pattern to remove the spiral phase front of the corresponding OAM mode, resulting in a high-intensity bright spot at the beam center which is separable from other OAM modes by spatial filtering.

### Experimental setup

**Figure 2:**
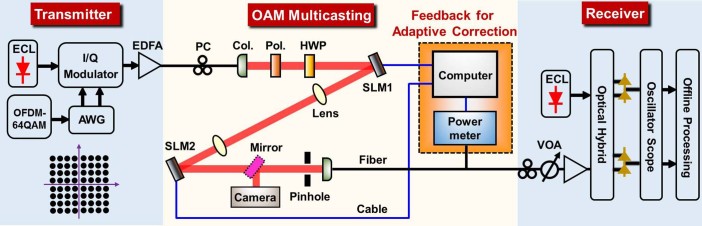
Experimental setup for feedback-assisted adaptive power-controllable OAM multicasting. ECL: external cavity laser; AWG: arbitrary waveform generator; I/Q Modulator: in-phase/quadrature modulator; EDFA: erbium-doped fiber amplifier; PC: polarization controller; Col.: collimator; Pol.: polarizer; HWP: half-wave plate; SLM: spatial light modulator; VOA: variable optical attenuator.

**Figure 3:**
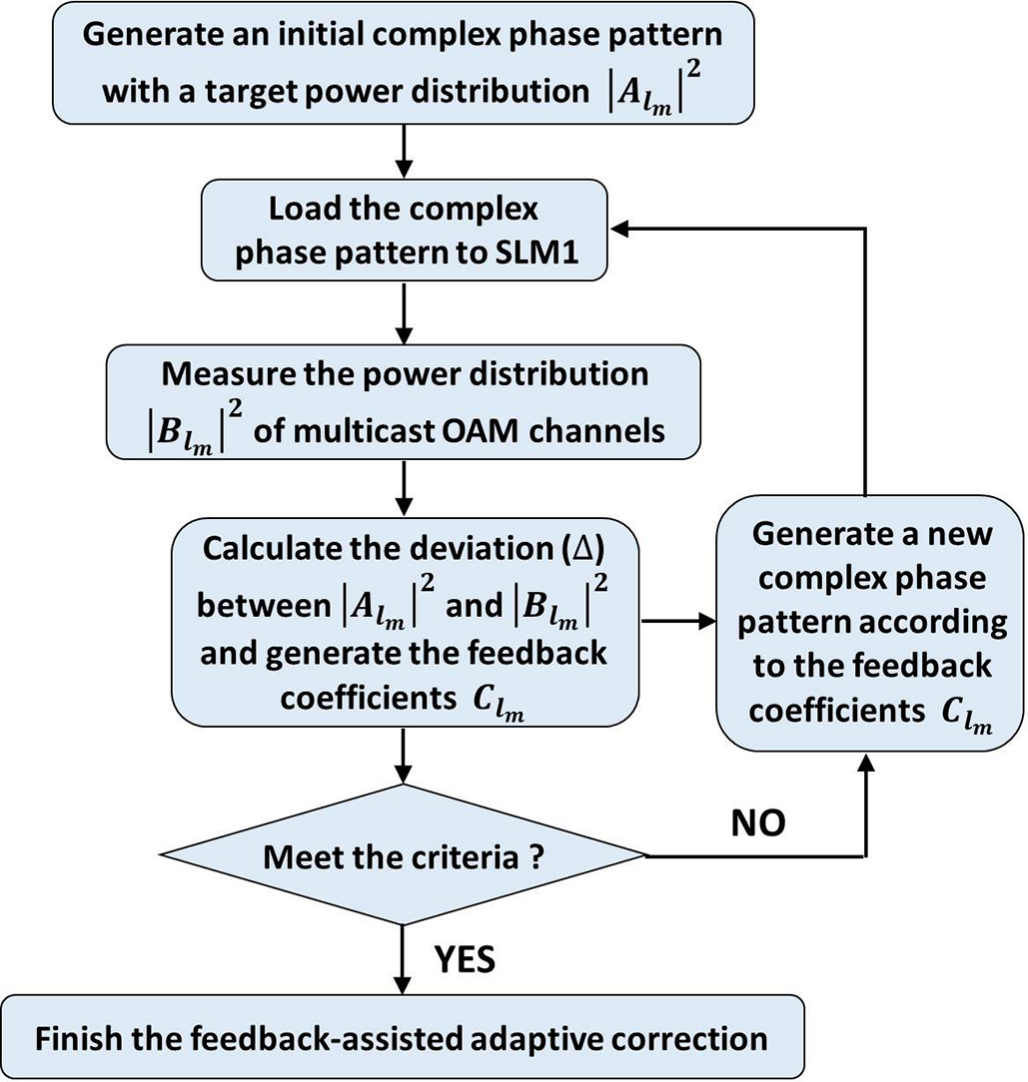
Flow chart of feedback-assisted adaptive correction.

The experimental setup for adaptive power-controllable OAM multicasting is illustrated in Fig. 2, including three parts named as transmitter, OAM multicasting and receiver. The transmitter part is used to generate OFDM64-QAM data signal at a wavelength of 1550 nm. The data signal through a single mode fiber is sent into a collimator to generate a data-carrying Gaussian beam in free space. The collimated Gaussian beam carrying OFDM 64-QAM signal is then reflected by the first spatial light modulator (SLM1) loaded with a complex multi-OAM phase pattern to generate collinearly superimposed OAM modes. Therefore, the energy of single input Gaussian beam is distributed onto multiple OAM beams (i.e. OAM multicasting). After free space transmission, another spatial light modulator (SLM2) is used to back convert one of multicast OAM modes (OAM*_l_*) to a Gaussian-like beam ( 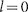) by loading the corresponding inverted single-OAM phase pattern ( 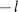). The back-converted Gaussian-like beam passes through a pinhole and is then coupled into a single mode fiber followed by adaptive correction and coherent detection. Remarkably, a feedback stage consisting of a computer and a power meter is employed in the setup to enable adaptive correction. Two spatial light modulators (SLM1, SLM2) and the power meter are connected to the computer. A MATLAB program manages the algorithms used for the generation of desired phase patterns loaded on SLM1 (complex multi-OAM phase pattern) and SLM2 (inverted single-OAM phase pattern). After generating multicast OAM channels by SLM1 and measuring the received power distribution (i.e. OAM spectrum) of OAM multicasting by SLM2 and power meter, the MATLAB program also computes deviations between the measured power distribution and the target one and returns feedback coefficients for adaptive correction. After the feedback-assisted adaptive correction, the OFDM 64-QAM data signal carried by the back-converted beam is sent to the receiver part shown in Fig. 2 for coherent detection. The flow chart of the feedback-assisted adaptive correction is illustrated in Fig. 3. *Step 1*: generate an initial complex multi-OAM phase pattern with a target power distribution of multicast OAM channels and load it on SLM1. *Step 2*: measure the actual power distribution by SLM2 and power meter and compare it to the target one. *Step 3*: calculate the deviation between the target power distribution and the measured one to be used for the generation of feedback coefficients. *Step 4*: determine whether the result meets the criteria. *Step 5*: if the result of *Step 4* is “NO”, a new complex multi-OAM phase pattern is generated according to the feedback coefficients obtained from *Step 3*. *Step 6*: repeat *Steps 2-5* until the result of *Step 4* is “YES” and the feedback-assisted adaptive correction is finished.

### Power-controllable multicasting of two OAM modes without feedback

**Figure 4:**
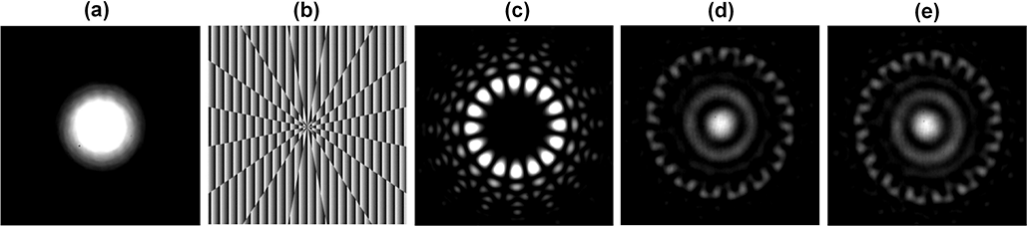
(a) Intensity profile of input Gaussian beam. (b) Complex pattern used to simultaneously generate OAM modes *l* = −8 and 8. (c) Intensity profile of superposed OAM modes *l* = −8 and 8. (d), (e) Back converted intensity profiles of OAM modes *l* = −8 and 8.

**Figure 5:**
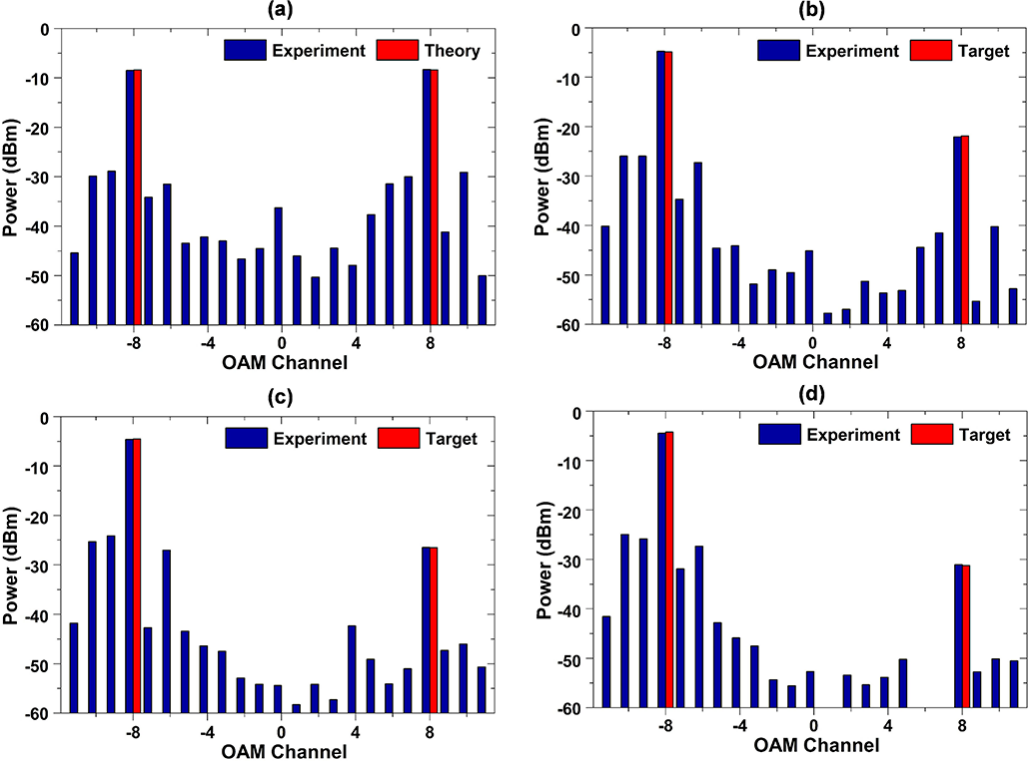
(a) Measured and theoretical power distributions for equalized power multicasting of OAM_−8_ and OAM_8_. (b)-(d) Measured and target power distributions for (b) 17-dB, (c) 22-dB and (d) 27-dB power difference multicasting of OAM_−8_ and OAM_8_.

We first explore the performance of power control for a two-OAM multicasting system. By using an adaptive-additive algorithm^20^ improved by a pattern search optimization algorithm, complex phase patterns are designed to multicast input Gaussian beam (Fig. 4(a)) to two OAM modes 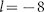 and 8. The power of each multicast OAM channel can be controlled by employing different weight coefficients in the generation of a complex two-OAM phase pattern. Fig. 4(b) shows a complex phase pattern used to generate two OAM modes with equalized power distribution. After loading the two-OAM phase pattern on SLM1, the input Gaussian beam is converted to the superposition of two OAM modes with a ‘petal’ intensity distribution, as displayed in Fig. 4(c). The ‘petal’ intensity is caused by the interference between OAM_−8_ and OAM_8_. When SLM2 is loaded with a specific OAM phase pattern ( 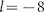 or 8), the corresponding multicast mode is back converted to a Gaussian-like beam having a bright spot intensity distribution at the center (shown in Fig. 4(d) and (e)). Then the central bright spot is filtered out by a pinhole and a single mode fiber and measured by an optical power meter. Fig. 5 shows the measured power distributions for different OAM channels.

In order to realize accurate power control, as mentioned above, we need to compute the power difference between target power and measured value and then generate a new complex phase pattern to correct the difference. At the beginning, we design a complex phase pattern to generate two OAM modes with equalized power. The measured and theoretical power distributions are depicted in Fig. 5(a). One can clearly see that the measured results are almost in agreement with the theory. The measured power of OAM_−8_ and OAM_8 _channels is −8.5 and −8.31 dBm, respectively, and the difference between them is −0.19 dB. Hence, we achieve relatively accurate power distribution even without any feedback correction in this two-OAM-mode multicasting situation. It means power control of simple two-OAM-mode multicasting can be entirely realized just by designing only once the complex phase pattern. In order to further prove this, we design a set of complex phase patterns with different target power distributions. In the experiment, the target power distributions are designed to be 17-, 22- and 27-dB difference between multicast OAM_−8_ and OAM_8_ modes. Fig. 5(b)-(d) show the measured results and target power distributions. The measured differences between multicast OAM_−8_ and OAM_8_ modes are 17.3, 21.9 and 26.6 dB, respectively. These measured power differences are close to the designed values. Consequently, for simple two-OAM-mode multicasting, just by controlling the power weight coefficients in the design of complex phase patterns, the power distributions of multicast two OAM (OAM_−8_ and OAM_8_) channels could be accurately controlled as expected.

### Adaptive power-controllable multicasting of six OAM modes with feedback

We have realized multicasting from input single Gaussian mode to OAM_−8 _and OAM_8_ modes with the power of each OAM channel controlled just by designing the complex phase pattern. The feedback correction is not necessary in that scenario because that OAM_−8 _and OAM_8_ have similar back-converted beam size resulting in similar receiving efficiency. However, in more complicated practical applications such as more multicasting OAM modes, it is difficult to accurately achieve the expected power distribution simply by once designing the complex phase pattern and the added feedback-assisted adaptive correction would be highly desirable to facilitate improved performance. Here we will show the experimental results for multicasting of six OAM modes with and without feedback.

**Figure 6:**
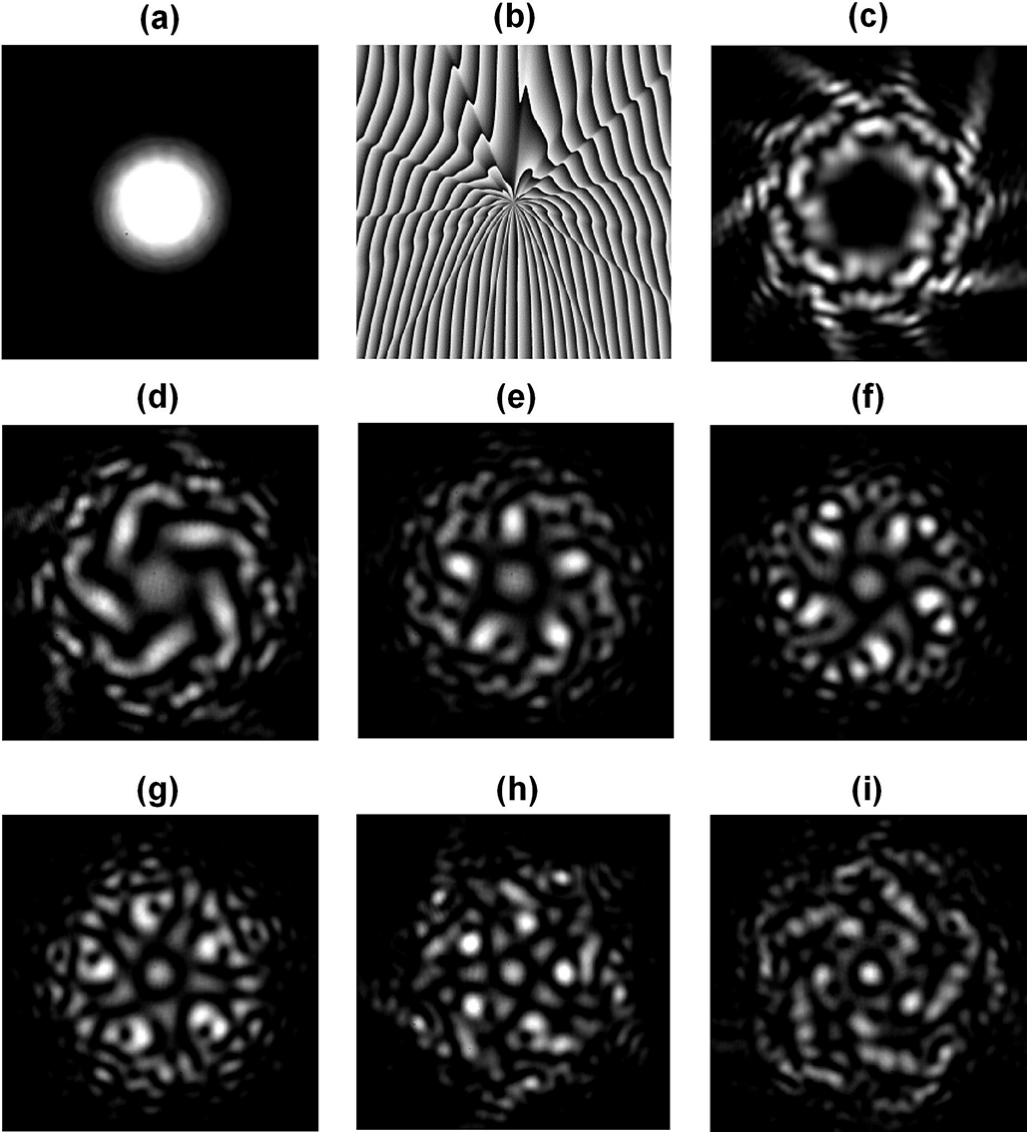
(a) Intensity profile of input Gaussian beam. (b) Complex phase pattern used to simultaneously generate OAM modes *l* = +5, +10, +15, +20, +25, +30. (c) Intensity profiles of superposed OAM modes *l* = +5, +10, +15, +20, +25, +30. (d)-(i) Back-converted intensity profiles of OAM modes *l* = +5, +10, +15, +20, +25, +30.

**Figure 7:**
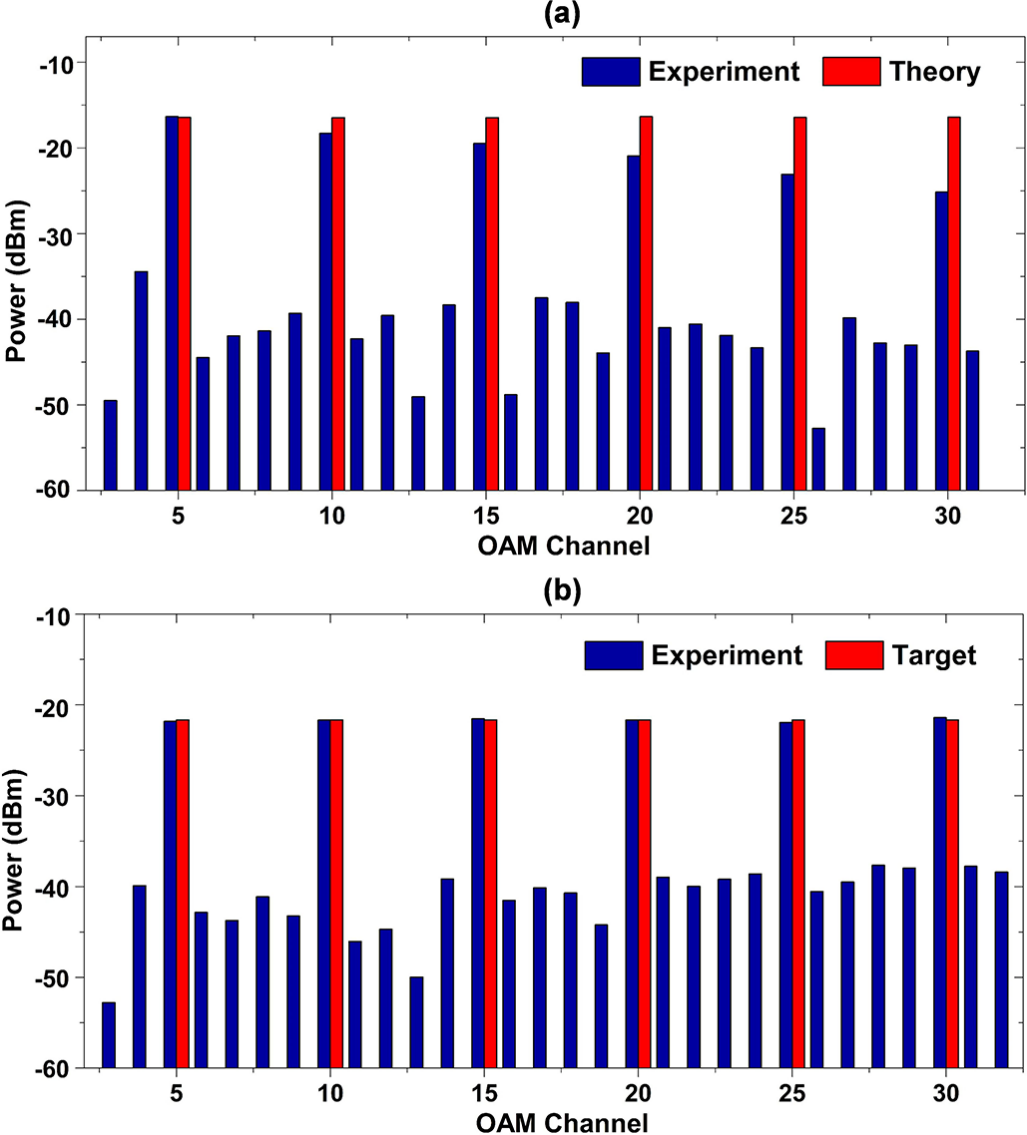
(a) Measured and theoretical power distributions for equalized power multicasting of OAM *l* = +5, +10, +15, +20, +25, +30 without feedback. (b) Measured and target power distributions for equalized power multicasting of OAM *l* = +5, +10, +15, +20, +25, +30 with feedback.

We generate a complex phase pattern to multicast input Gaussian beam to six OAM modes 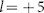, +10, +15, +20, +25, +30 with each mode having equalized power. Fig. 6(b) shows the complex six-OAM phase pattern for equalized power multicasting. After multicasting, the input Gaussian beam becomes the superposition of six OAM modes having a pentagonal dark center in the intensity profile, as shown in Fig. 6(c). In Fig. 6(d)-(i), we also show the back-converted beam intensity distribution of multicast OAM modes. One can clearly see the bright spots at the center after the back conversion from multicast OAM modes. The measured power distribution is plotted in Fig. 7(a). The phase pattern we designed is expected to distribute the same power in different multicast OAM channels, but the experimental results show that high-order modes have a lower power (e.g. OAM_+5_: −16.33 dBm, OAM_+30_: −25.14 dBm) compared to the theoretical design. This can be explained with the fact that different OAM modes have different spot sizes after back conversion to Gaussian-like beams. The larger the absolute value of 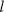 is, the smaller the spot size of OAM back-converted beam is, which can be clearly seen from Fig. 6(d)-(i). When coupled to a single mode fiber, different OAM back-converted beam size may have different coupling efficiency and cause the difference between the measured and target power distributions. In order to correct such deviation, feedback-assisted adaptive correction to the complex phase pattern is applied. A new complex phase pattern is designed based on the feedback and then loaded on SLM1 for pattern update. After feedback-assisted adaptive correction, the measured power distribution is depicted in Fig. 7(b). Compare to initial results in Fig. 7(a) without feedback, these results in Fig. 7(b) with feedback show favorable performance. The measured power of OAM 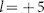, +10, +15, +20, +25, and +30 are −21.8, −21.7, −21.5, −21.7, −21.9 and −21.4 dBm, respectively. The difference of the maximum (−21.4 dBm) power and minimum (−21.9 dBm) power is only 0.5 dB which is much smaller than the result without feedback (~8.8 dB).

**Figure 8:**
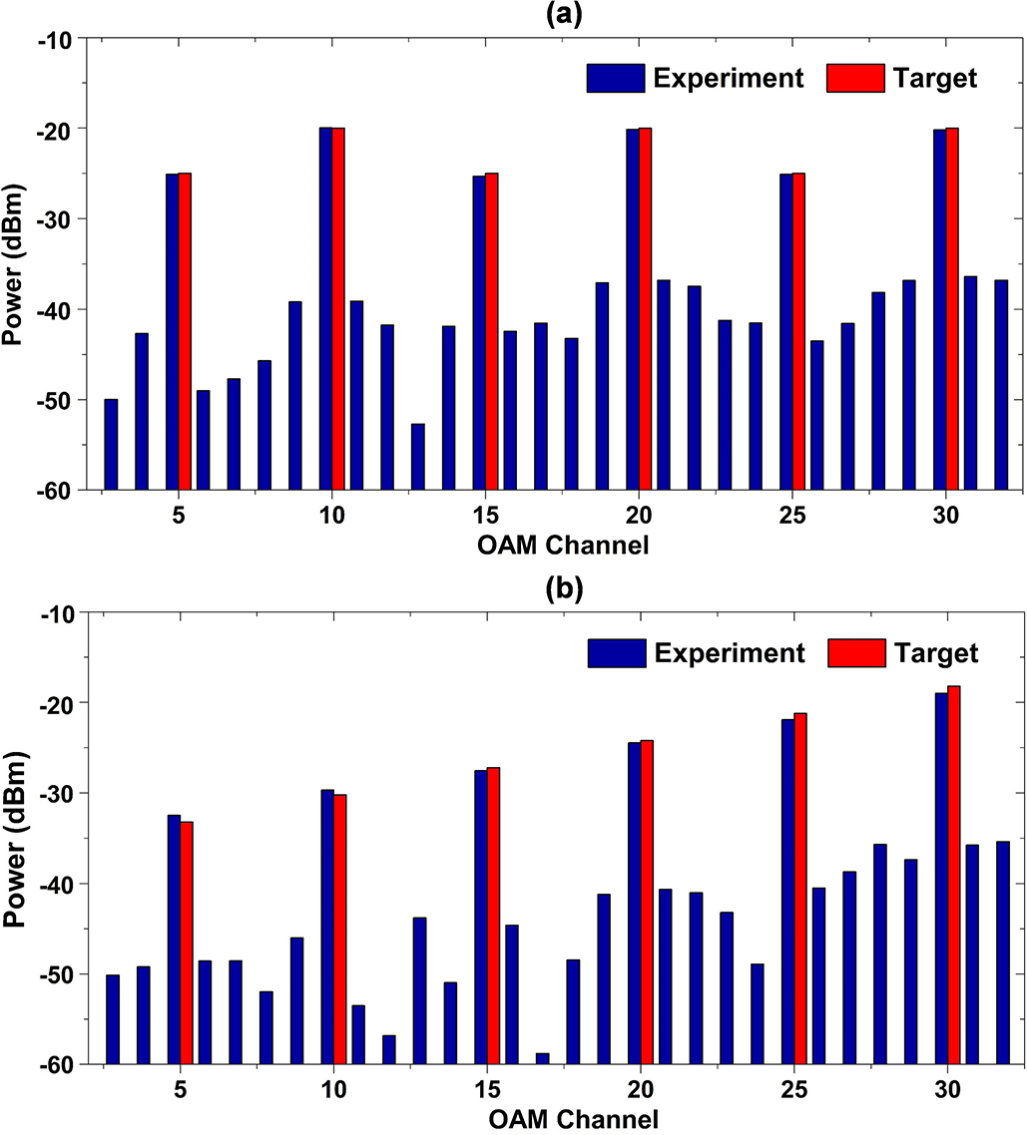
(a) Measured and target power distributions for “up-down” multicasting of OAM *l* = +5, +10, +15, +20, +25, +30 with feedback. (b) Measured and target power distributions for “ladder” multicasting of OAM *l* = +5, +10, +15, +20, +25, +30 with feedback.

To further demonstrate the feasibility of arbitrary power control, according to the feedback coefficient obtained from the equalized power multicasting, we design another two complex phase patterns with “up-down” and “ladder” target power distributions. The measured and target power distributions of “up-down” multicasting is shown in Fig. 8(a). The power of multicast channels alternate between low and high levels with a power difference of 5 dB. The measured power of back-converted OAM channel 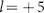, +10, +15, +20, +25, and +30 is −25.1, −20.0, −25.3, −20.1, −25.1 and −20.2 dBm, respectively. Compared to target values, the maximum power deviation is ~0.3 dB. Fig. 8(b) shows measured and target power distributions of “ladder” multicasting. The power of multicast channels increases with 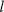 with a step power increase of 3 dB. The measured power of back-converted OAM channel 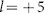, +10, +15, +20, +25, and +30 is −32.5, −29.7, −27.5, −24.5, −21.9 and −19.0 dBm, respectively. The maximum deviation between measured power and target value is assessed to be ~0.5 dB.

**Figure 9:**
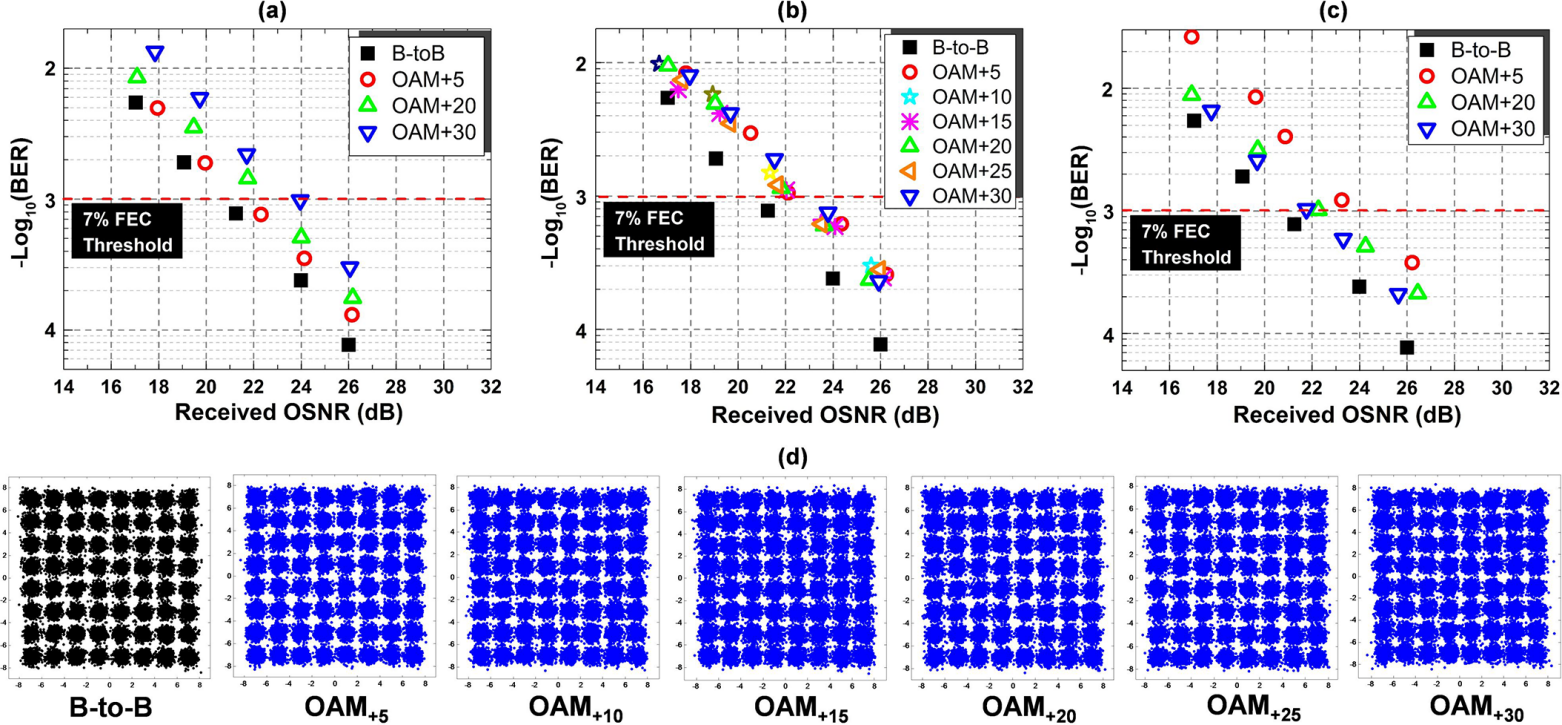
(a) Measured BER performance for equalized power OAM multicasting without feedback. (b) Measured BER performance for equalized power OAM multicasting with feedback. (c) Measured BER performance for “ladder” OAM multicasting with feedback. (d) Constellations for equalized power OAM multicasting with feedback corresponding to (b).

Finally, we measure the data-carrying performance of OFDM64-QAM signals over multicasting OAM channels. Fig. 9(a) shows the measured bit-error rate (BER) curves for OAM_+5_, OAM_+20_, OAM_+30_ in equalized power multicasting without feedback. The observed optical signal-to-noise ratio (OSNR) penalties at a BER of 1e-3 (forward error correction (FEC) threshold) are less than 1.0, 1.8 and 3.0 dB for the three multicast OAM modes, respectively. The relatively larger OSNR penalty of the higher-order OAM mode (OAM_+30_) is due to its lower receiving efficiency without feedback as shown in Fig. 7(a). The measured BER curves for all six OAM modes in equalized power multicasting with feedback is depicted in Fig. 9(b). It can be seen that the OSNR penalties at a BER of 1e-3 are less than 2.3 dB for all six channels. Moreover, Fig. 9(c) shows measured BER curves for OAM_+5_, OAM_+20_, OAM_+30_ in “ladder” multicasting with feedback. The observed OSNR penalties are less than 3.4, 1.9 and 1.4 dB at a BER of 1e-3 for OAM_+5_, OAM_+20_, OAM_+30_. The measured OSNR penalty decreases with the increase of OAM order. Such a phenomenon can be explained with the fact that higher-order OAM mode has larger received power in the “ladder” multicasting. Fig. 9(d) depicts typical constellations for equalized power OAM multicasting with feedback corresponding to Fig. 9(b).

## Discussion

We propose and experimentally demonstrate adaptive power-controllable OAM multicasting assisted by feedback correction. A single input Gaussian mode is multicast to multiple OAM modes by using a single complex phase pattern. For two-OAM-mode multicasting with similar beam size (e.g. OAM_−8_ and OAM_8_), the power of each OAM channel can be accurately controlled just by designing once the complex phase pattern. While for six-OAM-mode multicasting of OAM 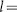 +5, +10, +15, +20, +25, +30, a feedback-assisted adaptive correction process is applied to realize arbitrary power control. The measured power distributions show good agreement with target power distributions. The precision of power control is less than 1 dB. Moreover, we measure the performance of OFDM 64-QAM signals over multicasting channels. For different power distributions, the BER curves and OSNR penalties are assessed showing favorable performance.

Owing to the highly-efficient iterative method, pattern search algorithm, and feedback-assisted adaptive correction employed in the experiment, it is possible to facilitate power-controllable OAM multicasting with superior performance using a single phase-only element. Several promising implementation effects are achieved.

- The performance of power-equalized OAM multicasting with multiple channels is improved, e.g. the difference of the maximum power and minimum power is only 0.5 dB for six-OAM-mode multicasting.
- In practical multicasting applications, different users might require different power budgets. A large power dynamic range up to 27 dB is achievable for power-unequalized OAM multicasting.
- Another distinct feature of the proposed OAM multicasting with feedback-assisted adaptive correction is its scalability to different kinds of practical systems with diverse receiving schemes. For instance, at the receiver shown in Fig. 2, the multicast OAM channel is back converted to a Gaussian-like beam which is collected by a single mode fiber. Generally, the feedback-assisted adaptive correction is robust to diverse receiving systems, i.e. the same setup could be used and the technique is fully compatible even when the configuration of the receiving system is changed from one to another according to different practical applications.

The presented adaptive power-controllable OAM multicasting, facilitating flexible and efficient OAM manipulation, may find wide potential use in future grooming OAM communications and networks.

## Methods

### Design of Phase-Only Element

For the generation of *n* collinearly superimposed OAM modes ( 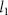, 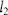, …, 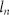), the mathematical description of the required transmission function is 


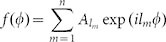
where 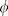 refers to the azimuthal angle, and the complex number 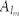 represents the target weight coefficient of each OAM mode. The ratio of power distribution is 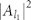: 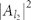: … 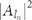. In general, such an 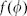 is not a phase-only function, i.e. 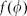 includes both phase and intensity modulation. To simplify the transmission function, a phase-only element having an approximate transmission function of 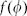 has been studied^21^,^22^,^23^,^24^. The objectives of designing the phase-only element are to ensure that the desired OAM modes get most of the power and keep the ratio of weights unchanged. We adopt the iterative method^21^ and improve it with pattern search algorithm^25^. An approximate phase-only transmission function with constant amplitude can be expressed as 


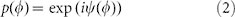



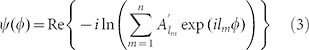
where Re{ } means 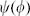 is a real value, and 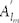 are tentative coefficients with initial values of 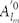. When using Fourier expansion, 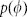 can be expressed as 


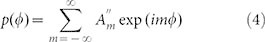
The difference between 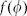 and 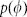 can be evaluated by a parameter of relative root-mean-square error, defined by 


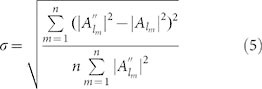
To realize high-performance OAM multicasting, a small 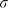 is desired. Then the problem becomes to find a suitable 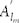 or 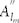 to minimize 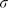. We use the pattern search algorithm to estimate the initial value 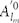 of the phase-only element, and then an iterative method is employed for minimization to find a favorable phase-only element.

### Feedback-Assisted Adaptive Correction

Remarkably, in a practical system, the ratio of actually received power distribution 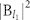: 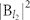: … 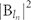 may somehow deviate from the target one 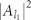: 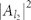: … 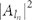 even though the phase-only element is well designed by the iterative method and pattern search algorithm. This can be explained with the fact that a practical receiving system (e.g. fiber coupling) may have different receiving efficiencies for different OAM modes (e.g. different OAM modes with different back-conversion spot sizes). So a feedback-assisted adaptive correction is highly expected as shown in Fig. 3. We define the feedback coefficients as 


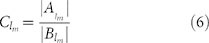
which are the ratio of the measured value ( 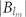) to the target one ( 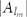). After getting the feedback coefficients, a group of new weight coefficients 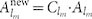 are obtained to generate an updated phase-only element with the target measured power distribution 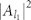: 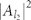: … 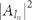 when taking the practical receiving system into consideration. Repeat the above process until the assessed deviation between the measured power distribution and target one meets the criteria (e.g. less than 1 dB).

## Author Contributions

**Author contribution statement** J.W. developed the concept. S.L. and J.W. conceived the experiments. S.L. carried out the experiments. S.L. and J.W. analyzed the experimental data. S.L. and J.W. contributed to writing and finalizing the paper.

## Additional information

**How to cite this article**: Li, S. & Wang, J. Adaptive power-controllable orbital angular momentum (OAM) multicasting. *Sci. Rep.* 5, 9677; DOI:10.1038/srep09677 (2015).
